# Challenges for impact evaluation of WHO’s normative output

**DOI:** 10.2471/BLT.24.292518

**Published:** 2024-10-01

**Authors:** Catherine Regis, Gaelle Foucault, Jean-Louis Denis, Pierre Larouche, Miriam Cohen

**Affiliations:** a University of Montreal, Faculty of Law, 3101, Chemin de la Tour, Montreal, H3T 1N8, Quebec, Canada.; b Institute of Health Policy, Management and Evaluation, University of Toronto, Toronto, Canada.

A clearer picture of the impact of the World Health Organization (WHO) normative work including guidelines, codes and regulations is needed.^1^^,^^2^ Member States and donors want their contribution to make an impact and WHO is committed to increased accountability.^3^^,^^4^ Yet, WHO acknowledges that normative work is often complex and assessing its impact may be more costly and challenging than conducting other types of evaluation.^5^ Therefore, developing stronger normative evaluation capacities that build on rich data and diverse methods within WHO and among Member States is needed.

Evaluating the impact of WHO norms requires appropriate data to be collected and made available. WHO relies on three main channels for evaluation data, but they underperform regarding normative impact. First, data can be collected through self-assessments, particularly regarding Member States' implementation of WHO norms. Although article 62 of the WHO Constitution requires Member States to report annually on compliance with WHO recommendations, these reports provide limited information due to lack of reporting standards and systematic compliance.^6^ More extensive data are collected through targeted self-assessments planned in the framework of a specific WHO norm. However, not all norms provide a reporting mechanism^1^^,^^2^ and self-assessments can vary in scope. For example, the States Parties Self-Assessment Annual Report tool standardizes the assessment by Member States of their capacities to monitor events relevant for the International Health Regulations (2005), while the assessment planned under the WHO Framework Convention on Tobacco Control is much broader because it is based on implementing the entirety of the convention. Furthermore, pointing to a national policy that reflects the WHO norm does not reveal whether the expected effects have been achieved by Member States and end users.^1^ Self-assessments provide a patchwork picture of normative impact.

Second, internal evaluations led by the WHO Evaluation Office or by WHO-commissioned experts provide additional data. Internal evaluations extend to sources beyond those found in self-assessments^7^ and may include end-user surveys, interviews or written submissions. Member States nonetheless remain key actors in the internal evaluation process since they approve future evaluations. Resources constraints force WHO to target its evaluations narrowly. For example, office-specific evaluations assess WHO's contribution at the country level and include one Member State at a time – for example Djibouti^8^ and Iraq.^9^ Nine country evaluations of this type have been conducted since 2017. Similarly, while the impact of WHO norms at the country level was the focus of a recent WHO report,^2^ time, methodological and financial constraints led to a limitation to seven specific country case studies and six norms. Limitations in scope hamper a comprehensive assessment of normative impact.

Third, external evaluations performed by academic and non-academic experts capture supplementary information related to the impact of WHO norms, which varies depending on the evaluation criteria and the research design. Since no prior authorization from Member States is required for these evaluations and more methodological exploration is feasible, evaluations can reveal different and more comprehensive information. Nevertheless, external evaluations face challenges regarding the uneven availability of WHO internal data and collaboration with key stakeholders. Without access to the WHO network, connecting with health ministries, WHO regional offices and other key actors for the evaluation can be difficult. As such, external research efforts remain scarce considering the complexity and cost of the task.^10^^–^^12^

These challenges are not insurmountable, but require WHO to increase its evaluation capacities. First, for normative evaluations WHO must strengthen its data strategy in relation to gathering, usage and openness. WHO must obtain a commitment from Member States to make impact evaluation a horizontal policy for all WHO norms. Doing so entails WHO-wide standardization of the data collection strategy. Such strengthening also implies the creation of an open-access database that regroups all implementation and evaluation processes on normative work from the three data evaluation channels. This database would give access to sources from which the data originated, and allow for targeted and comparative information search strategies.

Second, WHO should develop an evaluation strategy with partners to increase the evaluability of its normative function. Such joint strategy can be co-created by: (i) holding a conference of Member States and key partners to identify the objectives and content of the evaluation strategy; (ii) establishing a WHO Evaluation Office call for proposals to support the development and implementation of specific components of the strategy; and (iii) monitoring key milestones in the realization of the evaluation strategy. 

Through these internal and external measures, a culture of normative evaluation will develop in and around WHO, thereby improving the performance and accountability of the Organization and strengthening the commitment of stakeholders.
